# Durability and Long-Term Performance Prediction of Carbon Fiber Reinforced Polymer Laminates

**DOI:** 10.3390/polym14153207

**Published:** 2022-08-05

**Authors:** Eyad Alsuhaibani, Nur Yazdani, Eyosias Beneberu

**Affiliations:** 1 Department of Civil Engineering, College of Engineering, Qassim University, Buraidah 52571, Saudi Arabia; 2 Department of Civil Engineering, The University of Texas at Arlington, Box 19308, Arlington, TX 76019, USA; 3 Bridgefarmer & Associates, Inc., 2350 Valley View Lane, Dallas, TX 75234, USA

**Keywords:** carbon-fiber-reinforced polymer CFRP, environmental exposure, accelerated aging test, durability, Arrhenius method, ACI 440.2R

## Abstract

The feasibility of strengthening deteriorated or under-capacity concrete structures with external carbon-fiber-reinforced polymer (CFRP) laminates has been widely validated in the literature. However, there is a lack of knowledge on the in situ long-term performance and age-related environmental degradation of the mechanical properties of the laminates. The current study involved the immersion of coupons from a common new CFRP laminate in heated water at 23, 45, and 60 °C for 224 days. The coupons were then tested for residual tensile properties, such as tensile capacity and elastic modulus, using ASTM D3039 (2017) specifications. The CFRP tensile capacity and elastic modulus decreased by a maximum of 33% and 26%, respectively, for 224 days of exposure. Based on the test data, an age-based long-term prediction model with excellent reliability for CFRP laminate tensile capacity was developed. The model was then calibrated with test results from old CFRP coupons collected from an existing CFRP laminate retrofitted concrete bridge. The calibrated model output was then compared with the environmental reduction factor from ACI 440.2R-17 and a few other common sources. It was found that the ACI specified a reduction factor of 0.75, which does not consider the CFRP age and overestimates the design tensile strength of the CFRP laminate by approximately 13%, which may compromise the structural safety of retrofitted bridges. The reduction factors from the other guidelines varied between 0.51 and 0.85.

## 1. Introduction

According to the ASCE Report Card [1], the average age of highway bridges in the U.S.A. is 43 years and most of them are approaching or beyond their design lives. Therefore, an increasing number of bridges each year need rehabilitation or replacement and are unlikely to be replaced in a timely manner due to public funding limitations. Hence, there is increasing interest in fiber-reinforced polymer (FRP) composites for bridge repair and rehabilitation [2,3,4,5]. They have many advantages including high tensile strength, lightweight, corrosion resistance, and relative ease of application [6,7,8]. Although the feasibility of strengthening deteriorated concrete structures with FRP has been widely validated through laboratory experiments and field tests, there still exists a research gap in understanding its long-term durability and performance.

Numerous studies have been conducted to investigate the long-term performance of FRP composites using accelerated aging tests and predictive models, such as the Arrhenius method [9,10,11,12,13]. The durability of FRP is highly affected by moisture and high temperatures [14]. The evolution of CFRP materials, along with the process of immersion in different temperatures and for different durations, shows a continual decrease in the tensile strength as the exposure duration increases [15].

Li et al. [12] investigated the mechanical properties of CFRP and Glass FRP (GFRP) subjected to sustained load and environmental conditions including hygrothermal aging, freeze-thaw cycles, and wet-dry cycles. The sustained loads were 30% and 60% of the ultimate loads. The results showed that hygrothermal aging caused a significant decrease in tensile strength and elongation of the specimens with sustained loads. For the load-free condition, the wet-dry cycles aging test revealed a major decrease in tensile strength, but the tensile modulus showed excellent resistance to degradation. Silva et al. [13] studied the degradation of GFRP laminates under accelerated aging systems by immersing them in a saltwater solution at 30, 40, and 55 °C and salt fog cycles for a period up to 5000 h. The physical and mechanical properties of the specimens were then examined by moisture absorption and tensile tests. It was found that the laminate immersed in saltwater experienced a reduction in tensile strength. In addition, the specimen’s solution uptake and degradation increased with an increase in temperature.

Uthaman et al. [16] studied the aging of CFRP laminate upon immersion in water, acidic, and alkaline solutions at temperatures of 20, 40, and 60 °C. The mechanical and scanning electron microscopy results revealed that CFRP composites were more susceptible to the acidic solution and need protection to extend their service life. Karbhari and Abanilla [17] compared the Arrhenius and Phani and Bose methods’ long-term durability prediction performance by employing specimens immersed in a high-temperature water tank. It was found that the Arrhenius method is superior.

Homam et al. [18] studied the long-term durability of FRP subjected to various environmental conditions, such as freeze-thaw cycles, UV radiation, temperature variations, an alkaline environment, and moisture. Specimens were examined by direct tension and direct shear tests, which revealed that the mechanical properties of FRP are resistant to all the environmental conditions mentioned except moisture. Cromwell et al. [19] evaluated the performance of a CFRP plate, CFRP fabric, and GFRP fabric under nine different environmental conditions. The study then proposed reduction factors of 0.90, 0.50, and 0.80 for the three systems, respectively, which are appropriate for exterior exposure only. However, the investigation lacks actual data from the field for calibration purposes.

The environmental reduction factor for CFRP from ACI 440.2R-17 [20] was suggested to be 0.85, but no explanation was provided on how this factor was established, which led researchers to fill the gap by studying the long-term performance, durability, and resistance of CFRP to environmental conditions [13,15,17,19,21] through laboratory experiments. Yet, none of the results of these investigations were calibrated using data from an existing structure. Thus, to fill this knowledge gap, the current study used a prediction model employing short-term accelerated aging of CFRP under different environmental regimes using the Arrhenius method. Then, the model was calibrated by the results of long-term degraded samples extracted from an existing CFRP retrofitted bridge [22,23]. The calibrated model output was then compared with the environmental reduction factor from ACI 440.2R-17 [20] and a few other common international design guidelines. In the end, and based on the findings of this study, a function of design life to calculate the environmental reduction factor for CFRP laminate was proposed.

## 2. Experimental Program

### 2.1. Field Samples Collection

**Figure 1:**
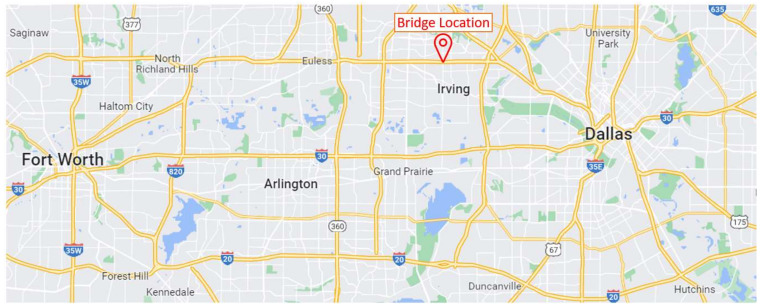
Bridge location map. Map data: Google © 2022.

**Figure 2:**
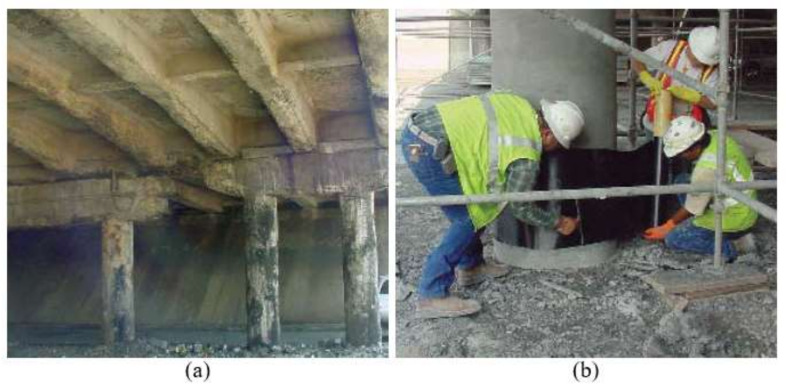
MacArthur Blvd. Bridge: (**a**) Post-fire; (**b**) during CFRP strengthening: Photos credit: Sika Corporation © 2006.

On 28 May 2005, the MacArthur Blvd Bridge (Figure 1) carrying State Highway 183 (SH 183) over MacArthur Boulevard in Irving, Texas was damaged by fire when a fuel tanker carrying 11.5 m^3^ of gasoline on SH 183 fell off the bridge and caught fire. The fire lasted approximately 30 min and caused severe damage to the concrete substructure and superstructure as shown in Figure 2a. It took 48 days to repair the bridge by chipping out the deteriorated concrete areas, cleaning the rebars, applying mortar to the removed areas, and strengthening several columns and girders using CFRP laminate (Figure 2b) [22,24]. On 16 June 2017, 12 years later, representative CFRP coupons were collected from the U-wrap of a girder in span 3 and were tested in the laboratory to obtain their tensile strength.

### 2.2. Field Samples Dimensions Preparation

After the field sample collection, four field CFRP coupons were prepared and cut to the suggested dimensions for the tensile test. To compare the performance of field samples, new samples of CFRP laminates using the same fiber and epoxy were created. Despite efforts to make the laminate thickness similar, there was a dissimilarity in the thinness between the new and field samples, which may be due to the automated saturation that was used during the initial field application [22,24]. To provide a logical comparison, the thickness and volume fraction of epoxy and fiber in the laminate had to be similar in both sample types. Equation (1) shows the relationship between the elastic modulus of laminate and the volume of laminate.

(1) $$E_{l}=\nu_{f}E_{f}+\nu_{e}E_{e}$$

where:

$\nu_{f}$ and $\nu_{e}$ = volume ratio of fiber and epoxy, respectively.
$E_{l}$, $E_{f}$, and $E_{e}$ = longitudinal elastic modulus of laminate, fiber, and epoxy, respectively.

Because the length and width of the new and field samples were identical, the volume of the fiber ($\nu_{f}$) and epoxy ($\nu_{e}$) were reliant only on the thickness of the fiber ($t_{f}$) and epoxy ($t_{e}$). The thickness of fiber ($t_{f}$), the thickness of laminate ($t_{l}$), and the elastic modulus of fiber$\;(E_{f}$) were known, which resulted in a reduction of Equation (1) to Equation (2), as follows:

(2) $$E_{l}=t_{f}E_{f}+(t_{l}-t_{f})E_{e}$$

The field samples’ laminate modulus of elasticity $(E_{l}$) was found from tensile testing. Assuming that the modulus of the fiber$\;(E_{f}$) did not change with time, the modulus of elasticity of the epoxy$\;(E_{e}$) was calculated as per Equation (2). Finally, the modulus of elasticity of the laminate was recalculated using the thickness of the laminate $(t_{l})\;$equal to the laboratory samples. This process provided a logical comparison between the field and laboratory samples as they had the same thickness and epoxy volume ratio.

### 2.3. Materials and Specimens, New Samples

The method chosen for the new samples was a typical CFRP wet-layup process. It consists of primarily woven unidirectional carbon fiber (SikaWrap Hex-117C made by Sika Corporation, Lyndhurst, NJ, USA) and epoxy (Sikadur 330 made by Sika Corporation, Lyndhurst, NJ, USA) made of two components with a mixing ratio of four-to-one by weight. Table 1 shows the properties of dry carbon fiber, epoxy, and laminate, which are identical to the materials used in the field samples.

**Figure 3:**
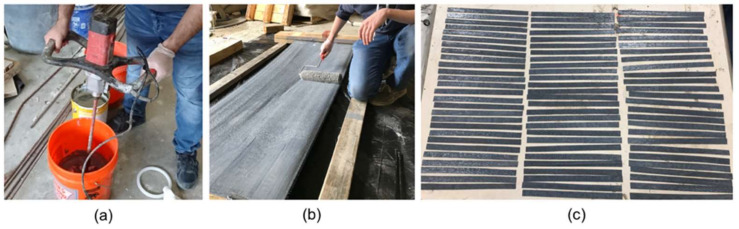
Preparation of the new CFRP coupons: (**a**) Mixing two-part epoxy resin; (**b**) applying the epoxy resin using a roller on both sides of the carbon fiber fabric; (**c**) cured laminates cut into strips (coupons).

**Figure 4:**
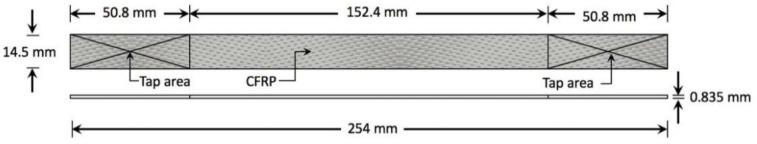
Geometry of new and field coupons.

The sample preparation involved the fabrication of a wooden frame to grip and stretch the carbon fiber. Then, the two-part epoxy was mixed thoroughly for 5 min using an electric mixer at a speed of 320 rpm at 23 °C, as shown in Figure 3a. The epoxy resin was applied using a roller and slipper on both surfaces of the carbon fiber ensuring full saturation, as shown in Figure 3b. The laminates were cured for three days at ambient temperature, and finally, coupons (Figure 4) were fabricated using an electric band saw.

### 2.4. Environmental Exposure, New Samples

**Figure 5:**
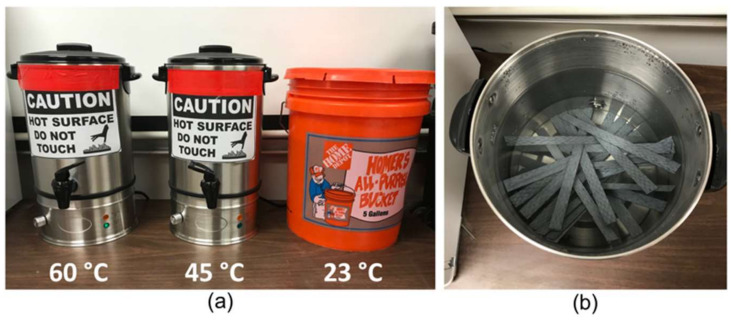
Accelerated aging: (**a**) Three different temperatures water tanks; (**b**) inside view of 45 °C water tanks with new CFRP coupons.

The environmental exposure protocol, adopted from a model proposed by Bank et al. [21], consisted of 80 specimens in an effort to investigate the degradation mechanisms of wet lay-up CFRP under various environmental conditions. The variables considered in the test were the exposure conditions and duration. To avoid reaching a viscous state of the CFRP, the specimens were conditioned to the typical 80% of the nominal glass transition temperature of most epoxies, or 60 °C [21]. The Arrhenius theory requires a minimum of three different temperatures to obtain degradation data [11]. Thus, after preconditioning, the CFRP samples were immersed in tap water at 23, 45, and 60 °C (Figure 5) for up to 224 days and were then removed for testing at intervals of 28, 56, 84, 112, and 224 days as listed in Table 2. The 23 °C samples were kept at room temperature with no applied heat. Water boiler tanks were used for the 45 and 60 °C exposure conditions with a temperature variation of ±3 °C. The current study used five samples for each exposure condition and duration plus five control samples, yielding a total of 80 specimens. Since the field samples were naturally aged, there was no need for them to be subjected to accelerated aging.

### 2.5. Tensile Test

**Figure 6:**
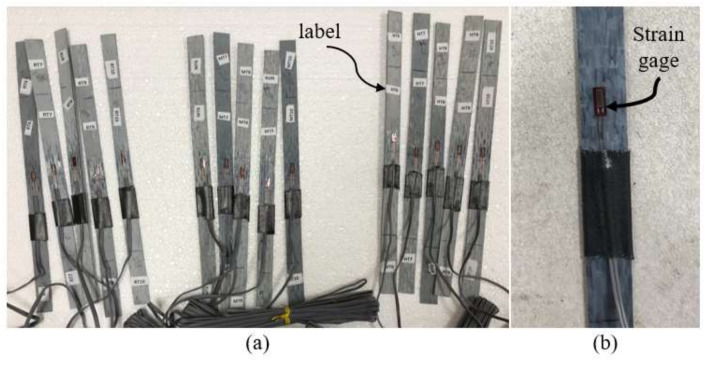
CFRP specimens: (**a**) Labeled specimens of each environmental condition; (**b**) closer view of CFRP specimen with strain gauge.

**Figure 7:**
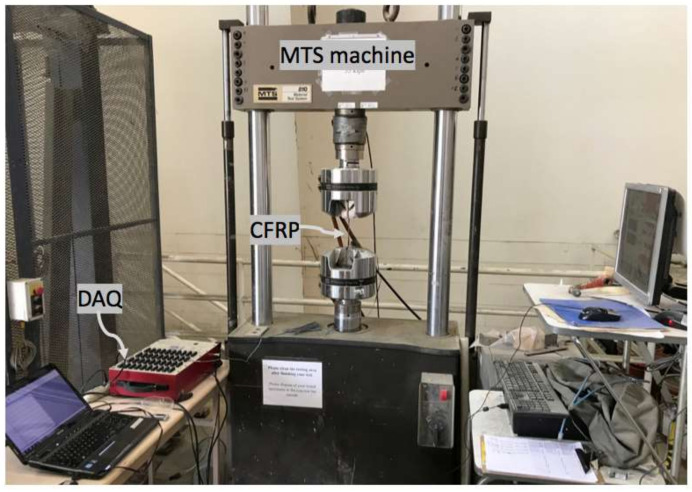
Tensile test setup showing the test apparatus.

**Figure 8:**
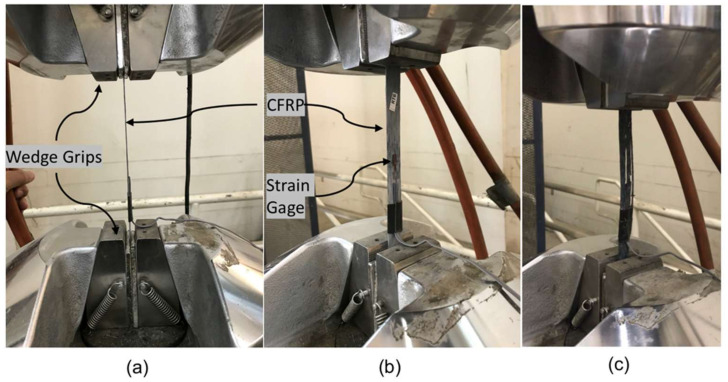
Tensile test set-up: (**a**) CFRP specimen with a straight alignment; (**b**) close view before tensile test; (**c**) failed sample.

**Figure 9:**
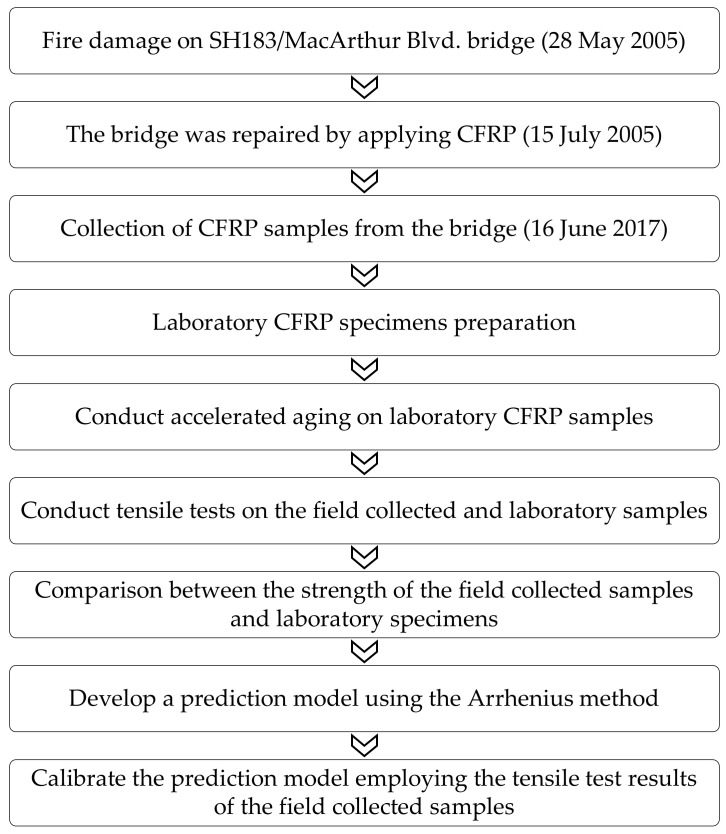
Summary of proposed work.

The new CFRP coupons were removed from the water tank after each specific period of exposure, after which their widths and thicknesses were measured with a 0.001-mm-resolution digital micrometer. A 350-Ω-resistance foil strain gage was installed at the mid-length of each specimen to capture their strain response (Figure 6). The coupons were tested per ASTM D3039 (2017) [25] employing the test setup shown in Figure 7 and Figure 8. The test was displacement controlled with a loading rate of 1 mm/min. Load, strain gauge, and displacement data were collected using a data acquisition system at a frequency of 8 Hz. An identical tensile testing procedure was used for the field samples. The flowchart shown in Figure 9 summarizes the experimental program.

## 3. Results

This section discusses the field sample results followed by the tensile strength, ultimate strain, and tensile modulus of new samples under different environmental exposure conditions.

### 3.1. Field Samples

The mechanical properties of the field and new samples were compared after applying Equation (2). For the same thickness of CFRP laminates, the field samples exhibited a 13% reduction in the elastic modulus. Moreover, the tensile strength of field samples was reduced by approximately 21%, which may be due to environmental conditions [22,24].

### 3.2. Tensile Strength

**Figure 10:**
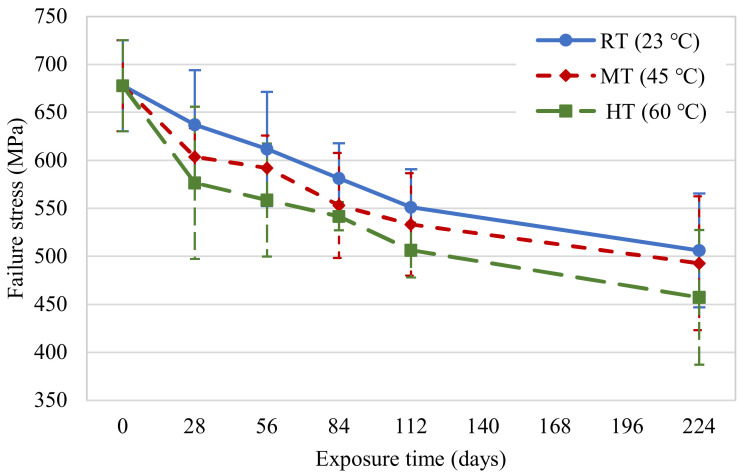
Tensile strength variations in new CFRP samples.

The evolution of the tensile strength of the new CFRP specimens for different temperatures and exposure durations is presented in Figure 10. A continual decrease in the tensile strength of the CFRP specimens occurred with an increase in the exposure duration, similar to the findings of past studies [15,26]. The tensile strength retention and coefficients of variation are shown in Table 3. Compared to their initial tensile strength, the new specimens experienced 25%, 27%, and 33% reductions on the 224th day for exposure conditions at 23, 45, and 60 °C, respectively. Clearly, water immersion at 60 °C significantly accelerated the degradation of tensile strength.

### 3.3. Strain Performance

**Figure 11:**
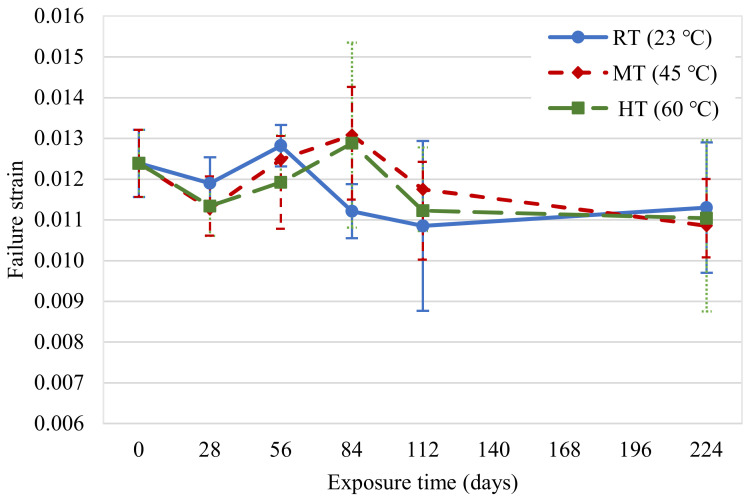
Failure strain variations in new CFRP samples.

The change in ultimate strain of the new CFRP specimens as a function of the exposure duration is illustrated in Figure 11. The strain does not show a consistent trend with an increase in the exposure duration, confirming the study by Xie et al. [15]. Compared to their initial failure strain, the specimens experienced 9%, 12%, and 11% reductions on the 224th day for exposure conditions at 23, 45, and 60 °C, respectively. The specimens immersed in 23 and 45 °C water experienced a 3% and 1% increase in failure strain, respectively, on the 56th day compared to their failure strain at the beginning of the exposure. This was attributed to the plasticization effect of the moisture ingress in the specimens [27].

### 3.4. Tensile Modulus

**Figure 12:**
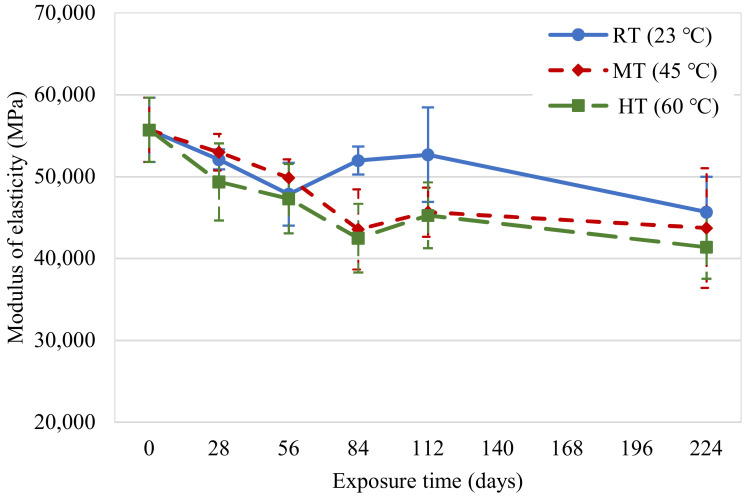
Tensile modulus variations in new CFRP samples.

A graph showing the variation of tensile modulus of the new CFRP specimens after exposure to elevated temperatures is presented in Figure 12. It predominantly exhibited a trend similar to the tensile strength. ACI 440.2R-17 [20] assumes that the modulus of elasticity of CFRP laminates is not affected by environmental conditions. Contrary to this, the specimens experienced 20%, 22%, and 26% reductions on the 224th day under exposure conditions of 23, 45, and 60 °C, respectively. A few past studies also showed similar findings [28,29,30].

## 4. Prediction Model of Long-Term Effects

Durability and service life predictions of FRP composites in civil infrastructures are challenging, as only short-term limited data are available. Due to the accurate results, the Arrhenius approach is widely used in such cases to show first-order effects and predict the service life of tested materials [12,15,16,17,21]. The degradation rate proposed by Nelson [31] is shown in Equation (3):

(3) $$k=A\cdot \exp\left(\frac{-E_{a}}{RT}\right)$$

where:

*k* = degradation rate (1/time).
*A* = constant of the material and degradation process.
$E_{a}$ = activation energy associated with the set of mechanisms.
*R* = universal gas constant (8.3143 × 10^−3^ kJ/mol K).
*T* = temperature (K).

The main hypothesis of the Arrhenius method is that the single dominant degradation mechanism does not change with temperature and time during exposure; however, the degradation rate accelerates with an increase in temperature. Equation (3) can be converted into Equations (4) and (5). In the former, the degradation rate, *k*, is expressed as the inverse of the required time to reach a given value of the material property. In the latter, the required natural logarithmic time for a property of a material to reach a given value is linearly related to 1/*T* with a slope of $E_{a}/R$.

(4) $$\frac{1}{K}=\frac{1}{A}\cdot \exp\left(\frac{E_{a}}{RT}\right)$$

(5) $$ln\left(\frac{1}{K}\right)=\frac{E_{a}}{RT}-ln\left(A\right)$$

### 4.1. Prediction Procedure

Following the procedure adopted by Chen et al. [10], the relationship between tensile strength retention, *Y*, of new CFRP laminates and exposure time was calculated from the experimental results of each exposure temperature as defined in Equation (6):

(6) $$Y=100\cdot \exp\left(\frac{-t}{\tau }\right)$$

where:

*Y* = percentage of tensile strength retention.
*t* = exposure time.
τ = 1/k = fitted parameter.

**Figure 13:**
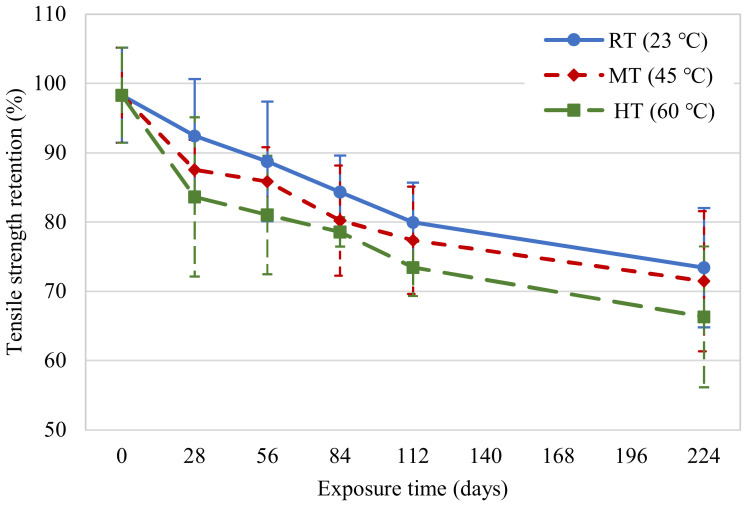
Tensile strength retention variations in new CFRP samples.

As shown in Figure 13, the relationship between tensile strength retention and the exposure time was obtained. By regression analysis, the fitted parameter, τ, and the correlation coefficients, *R*^2^, of the curves for each exposure temperature are summarized in Table 4, with the correlation coefficients, *R*^2^, of at least 0.88.

**Figure 14:**
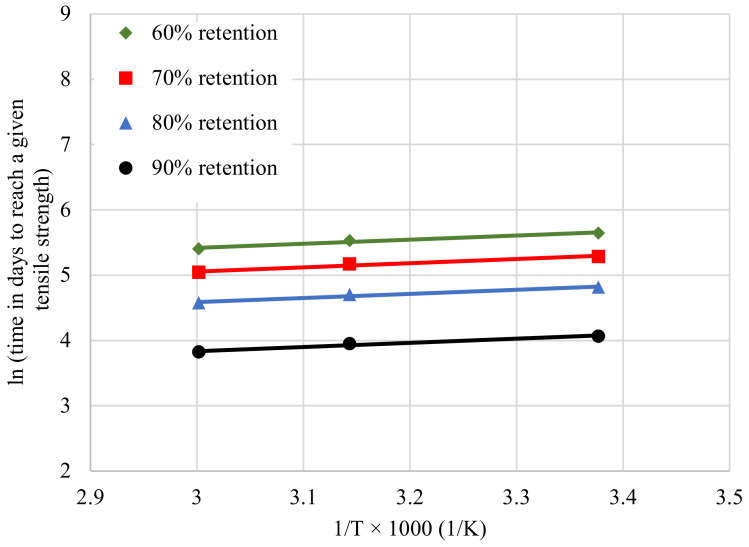
Arrhenius plots of tensile strength retention.

The Arrhenius relationships for tensile strength retention were obtained by plotting the natural logarithmic time to reach 60, 70, 80, and 90% tensile strength of CFRP laminates versus the inverse of the exposure temperature as shown in Figure 14. For a selected temperature on the *x*-axis, these lines provide the estimate of the time taken to reach the selected tensile strength retention values. Parallel straight lines were fitted to the data using Equation (5), and the slopes of the straight lines,$\;E_{a}/R$ resulted in correlation coefficients, *R*^2^, of 0.97 for 60, 70, 80, and 90% tensile strength retention. In order to meet the Arrhenius assumption, all temperature regression lines should have approximately equal slopes with correlation coefficients greater than 0.8 [21].

The time shift factor (TSF), initially used by Dejke [32], was based on the ratio between the time required for the property of a material to reach a certain strength retention value at two different temperatures. It can be expressed by Equation (7):

(7) TSF=t0t1=ck0ck1=k1k0=A⋅ exp−EaRT1A⋅ exp−EaRT0=expEaR1T0−1T1

where:

$t_{1}$ and $t_{0\;}$= required times for a property to reach a given value at temperatures of $T_{1}$ and $T_{0}$, respectively.
*c* = constant.
$k_{1}$ and $k_{0}$ = degradation rates at temperatures $T_{1}$ and $T_{0}$, respectively.

As stated, the field samples were collected from Irving, Texas. Thus, the reference temperature used in calculating the TSF was 18.92 °C, the mean annual temperature for that location [33]. The TSF values for each exposure condition are listed in Table 5.

**Figure 15:**
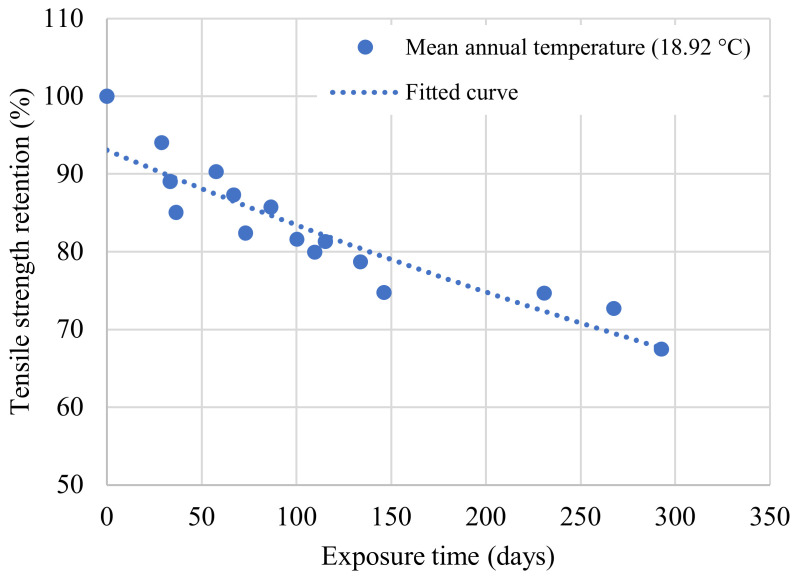
Tensile strength retention at the mean annual temperature (Irving, Texas).

The tensile strength retentions were then obtained by multiplying the exposure times at the three test temperatures with the corresponding TSF values, as shown in Figure 15. A curve was fitted to the data, yielding a correlation coefficient greater than 0.8, confirming the validity of this procedure [21].

A predictive equation, given by Equation (8), was employed to estimate the long-term tensile strength performance of CFRP using the Arrhenius method [12,17].

(8) $$f\left(t\right)=\frac{f_{0}}{100}\left[Aln\left(t\right)+B\right],\;\;(t>0)$$

where:

$f\left(t\right)$ and $f_{0}$ = performance attributes at time *t* (in days) and zero time, respectively.
A = constant denoting degradation rate.
B = material constant reflecting the early effects of post-cure progression.

**Figure 16:**
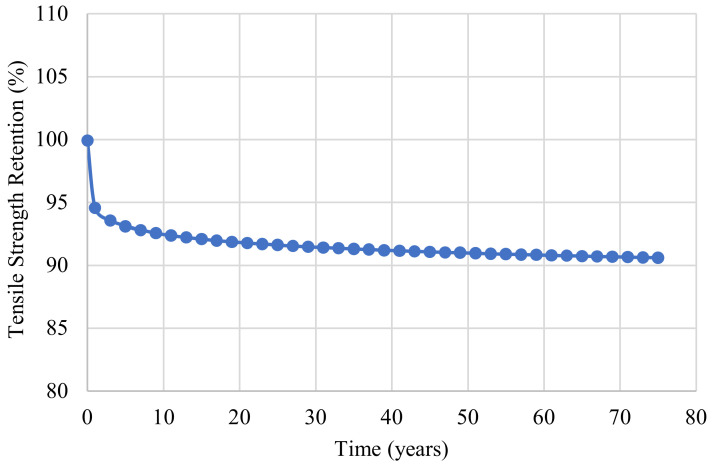
Prediction model based on the mean annual temperature for Irving, Texas.

A value of B equal to 100 indicates that the material is fully cured prior to environmental exposure. Employing regression analysis in Figure 15, the predictive Equation (9) for the mean annual temperature in Irving, Texas was developed and then used to plot Figure 16.

(9) $$f\left(t\right)=-0.92ln\left(t\right)+100$$

### 4.2. Prediction Model Calibration

**Figure 17:**
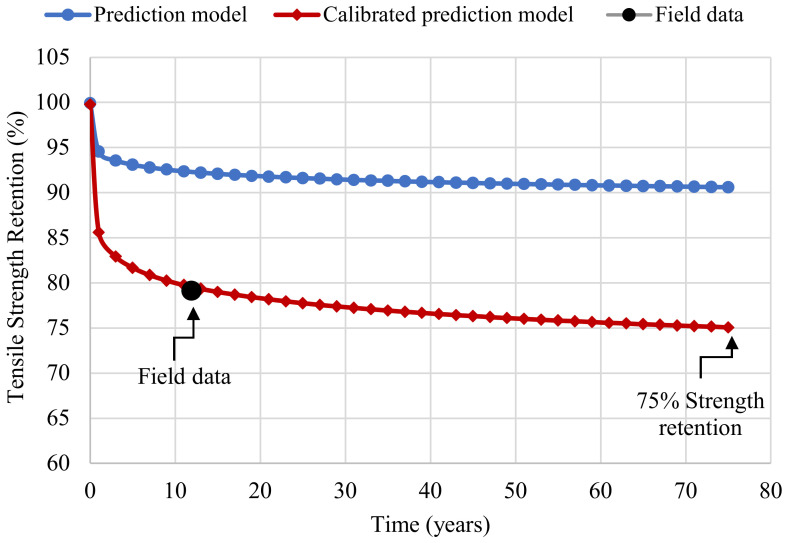
Calibrated prediction model.

It is essential to calibrate the new CFRP model with the actual field data after predicting the long-term behavior of the CFRP laminate. Thus, the predictive Equation (9) was modified to match the field data [22,23] by altering the degradation rate. Figure 17 shows the calibrated prediction model along with the uncalibrated model.

## 5. Discussion

In this section, the environmental reduction factors from five other international CFRP design guidelines were compared, and the new calibrated model was compared with ACI 440.2R-17 [20].

### 5.1. Environmental Reduction Factor

ACI 440.2R-17 [20] recommends that the CFRP laminate design’s ultimate strength, $f_{\mathrm{fu}}$, shall be determined by reducing the manufacturer’s reported strength, $f_{\mathrm{fu}}^{*}$, by the environmental reduction factor, $C_{E}$, as expressed in Equation (10). For the case of the CFRP laminate under exterior exposure, such as bridges, ACI prescribes an environmental reduction factor of 0.85.

(10) $$f_{\mathrm{fu}}=C_{E}\cdot f_{\mathrm{fu}}^{*}$$

where:

$f_{\mathrm{fu}}^{*}={\bar{f}}_{\mathrm{fu}}-3\sigma$.
${\bar{f}}_{\mathrm{fu}}$ = mean ultimate strength.
σ = standard deviation.

The UK code, TR55 (2012) [34], gives no explicit environmental reduction factors; rather, the characteristic material properties are divided by the factor of safety to determine the appropriate allowable design value,$\;f_{\mathrm{fd}}$, as defined in Equation (11).

(11) $$f_{\mathrm{fd}}=\frac{f_{\mathrm{fm}}-2\sigma }{\gamma_{\mathrm{mf}}\;\cdot \gamma_{\mathrm{mm}}\cdot \gamma_{\mathrm{mE}}\;}$$

where:

$f_{\mathrm{fm}}$ = mean ultimate strength.
$\gamma_{\mathrm{mf}}$ = partial safety factor for the strength of FRP.
$\gamma_{\mathrm{mm}}$ = partial safety factor for the method of manufacturing and application.
$\gamma_{\mathrm{mE}}$ = partial safety factor for modulus of elasticity of FRP.

For wet lay-up CFRP laminates, both $\gamma_{\mathrm{mf}}$ and $\gamma_{\mathrm{mm}}$ = 1.4, $\gamma_{\mathrm{mE}}=1.1$.

The Chinese code, GB 50608 (2010) [35], recommends calculating the allowable ultimate CFRP laminate design strength using Equation (12):

(12) $$f_{\mathrm{fd}}=\frac{f_{\mathrm{fk}}}{\gamma_{f}\;\cdot \;\gamma_{E}}$$

where:

$f_{\mathrm{fk}}=\mu_{f}-1.645\;\sigma_{f}$.
$\mu_{f}$ = mean ultimate strength.
$\sigma_{f}$ = standard deviation.
$\gamma_{f}$ = reliability index and brittle failure behavior of FRP materials.
$\gamma_{E}$ = environmental influence factor.

For wet lay-up CFRP laminate, the values of $\gamma_{f}$ and $\gamma_{E}$ = 1.4 and 1.2, respectively.

The European FIB Bulletin 14 [36] suggests calculating the CFRP laminate design tensile strength using Equation (13):

(13) $$f_{\mathrm{fd}}=\frac{f_{\mathrm{fk},}}{\gamma_{f,\mathrm{fib}}}\cdot \frac{\varepsilon_{fue}}{\varepsilon_{fum}}$$

where:

$\gamma_{f,\mathrm{fib}}$ = CFRP safety factor (1.35 for wet lay-up).
$\varepsilon_{fue}$, $\varepsilon_{fum}$ = effective and mean ultimate FRP strain, respectively.

The Italian [37] and the Egyptian [38] codes recommend environmental reduction factors identical to ACI 440.2R-17 [20].

### 5.2. Comparison of Calibrated Prediction Model with ACI 440.2R-17

The environmental reduction approach from ACI 440.2R-17 [20] was compared with the calibrated prediction model. According to AASHTO LRFD Bridge Design Specifications, the design life of highway bridges is 75 years [39]. The most conservative scenario of rehabilitation involves retrofitting a concrete bridge at the early stages of its design life, necessitated by impact damage from an over-height vehicle. As shown in Figure 17, the CFRP laminate has a 75% tensile strength retention after 75 years, which is 10% less than the environmental reduction factor from ACI 440.2R-17 [20]. This implies that ACI 440.2R-17 [20] overestimates the design tensile strength of CFRP laminates, which may compromise the structural integrity and safety of retrofitted bridges. Thus, the current study proposed Equation (14) to calculate an environmental reduction factor for CFRP laminate as a function of design life. If designers prefer to use a constant factor instead of a variable time-dependent factor, a factor of 0.75 is recommended, which corresponds to a design life of 77 years.

(14) $$C_{E}=-2.44ln\left(t\right)+100$$

where:

*C_E_* = environmental reduction factor.
*T* = design life (days).

### 5.3. Comparison of Calibrated Prediction Model with International Codes

Table 6 compares the environmental reduction factors from six widely used design guidelines. In order to facilitate the comparison, reciprocal safety factors of TR55 [34], GB 50608 [35], and Fib Bulletin 14 [36] were considered equivalent environmental reduction factors. Among these sources, TR55 [34] is the most conservative, proposing an environmental reduction factor of 0.51. Another study [40] also reported that the multiplicative factors specified by TR55 [34] can significantly reduce CFRP strength.

The second most conservative design guideline is GB 50608 [35], recommending an environmental reduction factor of 0.60. This underestimates the strength of the CFRP by 33%, as described by Li et al. [12], which may result in ineffective utilization of the CFRP. The European Fib Bulletin 14 [36] suggests an environmental reduction factor of 0.74. The Italian [37] and Egyptian [38] codes prescribe a value of 0.85, similar to ACI 440.2R-17 [20].

## 6. Conclusions

The current study developed a time-dependent prediction model for the environmental reduction factor of the tensile strength of CFRP laminates using the Arrhenius method and then calibrated it using CFRP coupon test results extracted from the existing CFRP retrofitted bridge.

Age and exposure temperature negatively affected the properties of new CFRP coupons. The immersion of CFRP laminates in water tanks with an elevated temperature resulted in the degradation of tensile strength, and the rate of degradation increased with an increase in the water temperature and age. The highest degradation was observed in 60 °C water for 224 days of immersion. The failure strain in the new CFRP laminates decreased with an increase in exposure time. However, the water immersion temperature had negligible or no effect, unlike the tensile strength. This may be attributed to the plasticizing effect of the moisture ingress in the specimens. The 12-year-old existing CFRP samples from an existing CFRP retrofitted bridge showed appreciable degradation in its mechanical properties. With identical thickness, the elastic modulus and the tensile strength of the field samples were 3% and 21% less than the new samples, respectively, most likely due to environmental degradation.

The study found that ACI 440.2R-17’s [20] specified environmental reduction factor of 0.85 for CFRP laminates is an overestimation of approximately 13% as compared with the factor of 0.75 from the developed predictive model. Therefore, using ACI 440.2R-17’s [20] specified factor results in overestimating the design tensile strength of CFRP laminates, which may result in an unsafe design. ACI 440.2R-17 [20] does not consider any environmental effects on the modulus of elasticity of CFRP laminates. However, the current study showed that the loss of the modulus of elasticity could be as high as 25%, depending on the exposure temperature and time.

The current study was limited to CFRP and did not consider factors such as freeze-thaw cycles, ultraviolet radiation, and immersion in alkali and acidic solutions. Thus, future investigations can further enhance this study by incorporating the stated limitations and developing prediction models for Glass and Aramid FRPs.

## Figures and Tables

**Table 1:** Properties of CFRP.

Material	Tensile Strength (MPa)	Young’s Modulus (GPa)	Failure Strain (%)
Dry carbon fiber	3793	234	1.5
Epoxy	33.8	4.5	1.2
CFRP laminate	724	56.5	1.0

**Table 2:** Experimental program for accelerated aging of new CFRP laminates.

Environmental Exposure	Duration (Days)	No. of Samples
Immersion in water at 23 °C (RT)	28, 56, 84, 112, and 224	25
Immersion in water at 45 °C (MT)	28, 56, 84, 112, and 224	25
Immersion in water at 60 °C (HT)	28, 56, 84, 112, and 224	25
Control samples (no exposure)	-	8
Total No. of samples		83

**Table 3:** Results from new CFRP coupons testing.

Environmental Exposure	Exposure Time (Days)	No. of Specimens	Average Failure Stress (MPa)	CV (%)	Tensile Strength Retention (%)
Unconditioned (Control samples)	0	8	678	6.99	100
RT (23 °C)	28	5	637	8.89	94
	56	5	612	9.72	90
	84	5	581	6.29	86
	112	4	551	7.17	81
	224	5	506	11.72	75
MT (45 °C)	28	4	604	4.95	89
	56	5	592	5.74	87
	84	5	553	9.90	82
	112	4	533	10.00	79
	224	5	493	14.16	73
HT (60 °C)	28	5	576	13.75	85
	56	5	559	10.55	82
	84	5	542	2.72	80
	112	4	506	5.65	75
	224	5	457	15.33	67

Note: RT, MT, and HT are room, moderate, and high temperatures, respectively; CV is the coefficient of variation.

**Table 4:** Correlation coefficients (*R*^2^) and fitted parameters for new CFRP tensile strength retention.

Water Temperature (°C)	Fitted Parameter, τ	*R* ^2^
23	554	0.99
45	494	0.94
60	434	0.88

**Table 5:** Time shift factors (TSF) for the mean annual temperature in Irving, Texas.

Environmental Exposure	Time Shift Factor
RT (23 °C)	1.02
MT (45 °C)	1.14
HT (60 °C)	1.22

**Table 6:** CFRP laminate environmental reduction factors.

Source	Country	Publishing Entity and Year	Environmental Reduction Factor
ACI 440.2R	USA	American Concrete Institute (ACI), 2017	0.85
TR55	UK	The Concrete Society, 2012	0.51
GB 50608	China	China Architecture & Building Press, 2011	0.60
Fib Bulletin 14	Europe	International Federation for Structural Concrete (Fib), 2001	0.74
CNR-DT 200	Italy	Advisory Committee on Technical Recommendations for Construction, 2014	0.85
ECP 208	Egypt	Egyptian Housing and Building National Research Center, 2005	0.85

## Data Availability

The data presented in this study are available on request from the corresponding author.
